# A Modified Dot-Pattern Moiré Fringe Topography Technique for Efficient Human Body Surface Analysis

**DOI:** 10.3390/s26031063

**Published:** 2026-02-06

**Authors:** Muhammad Wasim, Syed Talha Ahsan, Lubaid Ahmed, Subhash Sagar

**Affiliations:** 1 Department of Computer Science, UIT University, Karachi 75300, Pakistan; mwasim@uitu.edu.pk (M.W.); lahmed@uitu.edu.pk (L.A.); 2 Department of Electrical Engineering, UIT University, Karachi 75300, Pakistan; stahsan@uitu.edu.pk; 3 School of Computing, Macquarie University, Sydney 2113, Australia

**Keywords:** raster-stereography, moiré fringe topography, surface screening, spinal cord, trunk deformity

## Abstract

Raster-stereography and Moiré Fringe Topography are widely recognized as effective techniques for surface screening. Traditionally, these methods have been applied in various medical and clinical contexts, such as assessing human body symmetry, analyzing spinal deformities, evaluating scapular positioning, and predicting trunk-related abnormalities. Both techniques have proven to be reliable tools for examining the human body surface and identifying health-related issues. However, in these techniques, line grids projected onto non-uniform surfaces often break or distort, complicating curvature detection. Capturing and digitizing these distortions through photographymeans further reducing accuracy due to low contrast between background and projected lines. In this paper, we present a modified, i.e., dotted-based, approach to Moiré Fringe Topography construction, offering a simpler, more accurate, and efficient method for recording human body surface curvatures. The proposed technique significantly reduces the complexity of the data acquisition process while maintaining precision in surface analysis. A Single-Photon Avalanche Diode (SPAD) image sensor was used to capture the Moiré patterns.

## 1. Introduction

Both raster-stereography [1,2,3] and the Moiré Fringe topography [4] are widely used, ionization-free, and 3D surface profiling techniques. Raster-stereography operates by projecting a line grid onto an object, where distortions in the grid indicate underlying surface curvatures. Moiré fringe topography, on the other hand, exploits optical interference patterns produced by finely spaced grids, traditionally implemented using wire or thread arrays with sub-millimeter spacing [4]. The conventional Moiré fringes are formed as a pattern of interference lines generated by the superposition of two sets of parallel lines or grids which are marginally inclined relative to each other. The grid line width should be equal to the spacing between adjacent lines. Both methods have been applied extensively to human body analysis, including the assessment of spinal deformities, evaluation of back symmetry, detection of scoliosis, trunk abnormality analysis and facial curvature studies [1,2], as explained in Section 2.

Although effective, these techniques face several limitations. In raster-stereography, line grids projected onto non-uniform surfaces often break or distort, complicating curvature detection. Capturing and digitizing these distortions through photographic means further reduces accuracy due to low contrast between background and projected lines. Similarly, moiré fringe topography is sensitive to illumination conditions and prone to fringe overlap, making interpretation difficult. During experimental trials conducted in the Image Processing Research Lab, it was observed that in conventional Moiré Fringe Topography, the extraction of Moiré patterns from recorded projections becomes challenging when applied to human faces or bodies. This difficulty arises due to color and intensity similarities between the projected fringe patterns and the surface of the object, which often cause fringe discontinuities and breakages. As a result, accurate curvature extraction becomes problematic (as discussed in Section 5). These challenges restrict clinical usability and reduce reliability in precision-demanding contexts.

To address these limitations, this study introduces a novel Circular-Dotted moiré technique. Unlike traditional continuous fringe patterns, the proposed approach employs a dotted projection pattern that effectively eliminates the issue of pattern discontinuity while traversing complex surfaces such as human faces. This technique enables accurate capturing and recording of surface curvatures with improved robustness. Furthermore, the proposed method demonstrates faster computational performance, enhanced reliability in terms of accuracy, and reduced algorithmic complexity compared to conventional moiré fringe topography. It further enables 3D mapping of the subjects specially for human faces without the use of X-rays or any other ionizing radiation that could be harmful.

The technique was used and processed to analyze various 3D objects and human faces using the developed algorithms. Thus, the contribution of this work lies in providing a simplified, accurate, safe and clinically viable alternative to conventional surface topography methods, advancing non-invasive human body surface analysis. To record Moiré patterns on human faces and objects in low-light conditions, the authors used a *1-megapixel* single-photon avalanche diode image sensor in Image Processing Research Lab (IPRL). This sensor enables the camera to capture highly detailed images even at one-tenth of the typical illumination, making it suitable for dark environments.

## 2. Literature Review

Raster-stereography and Moiré Fringe Topography have been widely recognized as effective techniques for 3D surface analysis. These methods are non-invasive, non-contact, nondestructive, and safe for human applications, making them particularly suitable for clinical diagnostics and biomechanical studies [1,2,3]. Several studies have explored their utility in medical contexts.

The surface topography technique is now a radiation-free option for screening and monitoring the outer trunk shape in healthcare. Current studies have brought together the methods of raster-stereography and Moiré, focusing on how they can be applied for repeated analysis [5]. This background is very important to this research because it shows that surface screening is clinically valuable but often limited by issues with data collection and processing. Specifically, non-uniform anatomy or reflectance can affect how well projected patterns are read or how reliable the reconstruction is in real human scans [5]. Therefore, we propose a Moiré fringe pattern based on dots to improve curvature recording and simplify data collection for body surface analysis.

Current surface topography research is increasingly linking surface capture to automated interpretation for screening decisions, going beyond measurement. To measure trunk asymmetry for scoliosis screening, Mohamed et al. present a 3D markerless surface topography pipeline with convolutional neural networks [6]. Their results show that stable, high-quality surface representations that are consistent across subjects and sessions are necessary for accurate evaluation. This is especially important when complex anatomy leads traditional line-grid projections to degrade, creating unclear features that introduce errors in further analysis [6]. Therefore, the current work focuses on Moiré patterns that are ready for analysis and have simpler acquisition requirements, supporting both computational processing and screening workflows.

Recent research shows that digital-first Moiré processing can be redesigned to improve the speed and reliability of topography measurement [7]. Chen et al. introduce a binocular vision-based 3D sampling Moiré technique that maintains accuracy while simplifying phase matching and boosting computational efficiency. Since the Moiré pipeline can be set up for effective reconstruction instead of being seen only as an outdated optical method, this approach aligns with the current contribution [7]. Furthermore, it helps meet the design goal of reducing the complexity of acquisition and digitization while keeping sensitivity to surface deformation and curvature. Building on this idea, the proposed dotted-based Moiré construction focuses on line grid failures on uneven human surfaces.

Moiré profilometry’s recent developments reduce the need for filtering steps. This change can improve fringe quality and increase flexibility in real-world imaging situations [8]. Zhang et al. use two complementary deformed stripe patterns for spatial computer-generated Moiré profilometry, allowing for the extraction of Moiré fringes in the spatial domain [8]. Fewer captures and more reliable fringe retrieval can enhance tolerance to posture changes and small movements. This is important for scanning the human body. Moreover, minimal-shot processing simplifies data collection. This is especially important in highly curved areas of the body, where projected structures can break or distort. To improve continuity in complex anatomy, the current study replaces fragile line structures with dot-based encoding. This aligns with the growing focus on stronger Moiré processing.

Dot-pattern structured light is particularly relevant because it supports fast acquisition under motion sensitivity and high-throughput constraints. In their review of high-speed 3D vision based on structured light, Miyashita et al. emphasise the importance of pattern design in determining processing feasibility and robustness [9]. Dot encoding is appealing for non-uniform human surfaces because it reduces the “broken-line” issue that continuous grids have and maintains usable features in the face of sudden curvature changes. This explains the current decision to create Moiré information from dotted structures, which enhances contrast and robustness for photographic capture when compared to line grids. This design decision is also in line with the suggested system’s use of a SPAD image sensor for Moiré capture, where separable features can support accurate pattern acquisition.

The case for dot-pattern selection is further strengthened by recent optical hardware, which shows efficient reconstruction and information-rich dot projection with compact form factors. Shen et al. describe a baseline-free structured-light method that combines a simple and quick reconstruction technique with a metasurface double-helix dot projector. Accurate point-cloud acquisition for actual scenes, such as a living human face, is one of their demonstrations that show viability on biologically complex surfaces [10]. These findings lend credence to the idea that dot arrays can facilitate effective reconstruction without relying too heavily on continuous line integrity. In order to improve curvature recording and lower acquisition complexity in human body surface screening, the suggested dotted-based Moiré fringe topography uses dot-encoding.

The Scoliosis Assessment App, based on surface topography technology, provides an accurate, accessible, and radiation-free method for detecting clinically significant scoliosis [11]. The authors claimed that the proposed system serves as an effective tool for topographic analysis, providing objective measurements of torso shape that complement existing imaging modalities. It enables reliable assessment of clinical parameters, supporting objective evaluation of symmetry, body shape before and after surgery, and longitudinal tracking of pathology—all without exposure to ionizing radiation [12]. Diagnostic evaluation can be performed using non-radiological, non-contact, non-invasive methods.

As far as raster-stereography is concerned, ref. [1] investigated the validity and consistency of raster-stereography in assessing patients with adolescent idiopathic scoliosis (AIS), demonstrating its potential as a reliable alternative to radiographic methods. Similarly, ref. [13] examined the application of raster-stereography for monitoring scoliosis progression and related trunk abnormalities, highlighting its role in long-term follow-up. Further advancements in raster-stereography include its use for high-precision 3D back surface analysis [14]. This work emphasized accuracy in reconstructing spinal geometry, particularly in cases of idiopathic scoliosis treated with anterior correction and fusion. Ref. [15] presented a study of foot posture variations and spinopelvic mechanics using 4D rasterstereography. Thus, consistency of raster-stereography-based surface reconstruction for both pathological and healthy subjects has been confirmed, reinforcing its reliability in clinical practice.

Moiré Fringe Topography is a 3D imagining technique that is not only non-contact but also non-invasive and is widely utilized for medical purposes and in industry to sense slight fluctuations in surface gradients. Several researchers have proved that the changes in the backs of the patients suffering from scoliosis can be measured using Moiré frames. The ability of Moiré to yield graphical data instantaneously and accurately makes it an ideal tool for outlining the surface of the back and relating it to back deformities. Moiré Fringe Topography offers a promising alternative to radiography for assessing spinal and trunk deviations.

This study [4] aimed to develop and validate an assessment tool for a Moiré topogram generated specifically using the Shadow Moiré technique of the dorsum [16]. In [17], the technique was employed to analyze human foot morphology, while this method has since been widely for the detection, quantification, and longitudinal follow-up of trunk deformities, posture assessment, gait analysis [3,18,19,20,21] and the study of neurological disorders [22].

Collectively, these studies establish raster-stereography and Moiré Fringe Topography as robust surface screening tools with broad applications in human body analysis. However, challenges related to measurement accuracy, surface reconstruction, and interpretability remain, motivating the need for further methodological improvements.

## 3. Raster-Stereography and Modification in Construction

The classical concept of raster-stereography [1,2] employs a raster grid composed of horizontal and vertical lines, forming a matrix of small squares. When this grid is projected onto a non-uniform surface using a multimedia projector, distortions occur in the square patterns, providing curvature information of the underlying object. By applying mathematical models, decision parameters can be derived from the coordinate deviations of the distorted lines, enabling surface characterization and recognition across different objects.

Let ($x_{o}$,$y_{o}$) denote the reference grid coordinates on a flat calibration plane, and ($x^{\prime }$,$y^{\prime }$) the observed distorted coordinates on the surface under study. The displacement vectors are expressed as follows:

(1) $$\Delta x=x^{\prime }-x_{o}\;\;\;\Delta y=y^{\prime }-y_{o}$$

From these distortions, the local curvature C(x,y) may be estimated as follows:

(2) $$C\left(x,y\right)=\sqrt{{\left(\Delta x\right)}^{2}+{\left(\Delta y\right)}^{2}}$$

For full three-dimensional reconstruction, triangulation principles are applied. If the projector and camera are separated by a known baseline distance B, and the camera is calibrated with focal length *f*, the depth *Z* of a surface point can be estimated from the disparity *d*:

(3) $$Z_{x}=\frac{f.B}{|x^{\prime }-x_{o}|}$$

Similarly,

(4) $$Z_{y}=\frac{f.B}{|y^{\prime }-y_{o}|}$$

(5) $$Z\left(x^{\prime },y^{\prime }\right)=\sqrt{{\left(Z_{x}\right)}^{2}+{\left(Z_{y}\right)}^{2}}$$

Each surface point can thus be represented in 3D space as follows:

(6) $$P\left(x,y,z\right)=(x^{\prime },y^{\prime },Z\left(x^{\prime },y^{\prime }\right)$$

The collection of reconstructed points forms a dense 3D point cloud, from which curvature maps and diagnostic parameters may be extracted. In the work presented in this paper, the curvature has been calculated using Takasaki’s equation [23] discussed in the next section.

**Figure 1:**
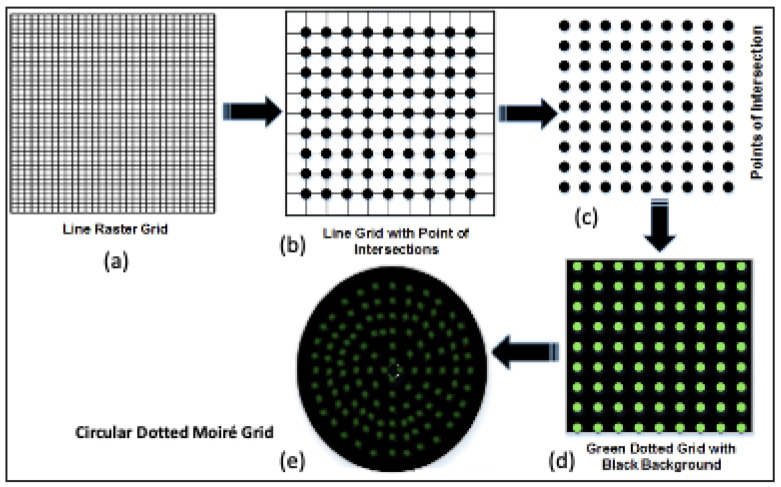
(**a**) Conventional line raster grid, (**b**) line grid with points of intersection, (**c**) array of dots, (**d**) dotted raster grid with black background, and (**e**) circular dotted grid.

However, experimental evaluations conducted at IPRL revealed a critical limitation in the conventional line-based raster grid. The line raster technique had valid curve tracing issues due to breaks in the line. Specifically, poor contrast between the wooden surface and projected lines frequently caused line discontinuities, as illustrated in Figure 1a. These discontinuities hindered accurate tracing of real surface curves, reducing the reliability of curvature analysis.

To overcome these limitations, a modified dotted raster-stereography technique was introduced. In this approach, dots were placed at each intersection point of the raster grid, replacing continuous lines. As shown in Figure 1b–d, the dotted grid was projected onto a black background, which significantly improved point detection accuracy and minimized contrast-related issues (Table 1). This modification enhanced the precision of curvature extraction and facilitated more robust surface recognition. The proposed dotted raster grid was initially applied by the authors to human face recognition tasks, where it demonstrated superior performance compared to the traditional line-based method.

**Figure 2:**
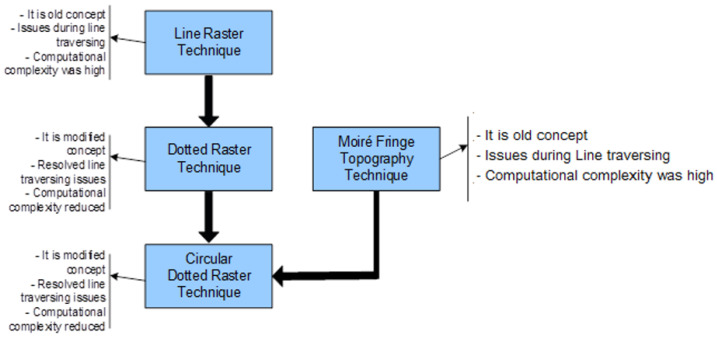
Framework of the modified techniques.

Figure 2 provides the framework of the conventional line-raster grid, and the proposed concepts of modified dotted and circular-dotted techniques; the last one is proposed to be used in the modified moiré fringe topography mechanism, as explained in the next section.

## 4. Conventional Moiré Fringe Topography Technique

**Figure 3:**
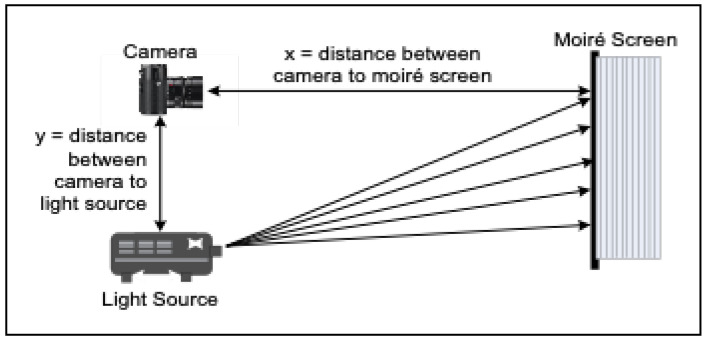
Principle of conventional (shadow) moiré topography.

Figure 3 depicts the concept of shadow moiré analysis, in which light passes through a grid from a source and falls behind the shadow stripes on the surface. An observer perceives light initiating from certain zones while watching through the same grid. As a result of optical processing, map-like contours are created by moiré topography.

The design includes a system that includes a light source, grid and camera as directed by the Takasaki equation [23] and as mentioned in Table 2.

(7) $$h_{n}=\frac{nx}{\frac{y}{p}-n}$$

$h_{n}$ is derived, using Equation (8), from the points on the boundary of the shadow behind the screen to the screen distance. The distance between the intervals of fringe and shadow planes differs as a function of geometry.

(8) $$\Delta h=\frac{xy}{p\left[\frac{y}{p}-\left(n+1\right)\right]\left[\frac{y}{p}-n\right]}$$

The moiré fringe topography technique required a specific hardware setup to record moiré patterns. The construction of a large wooden frame, which consisted of a large number of vertical fishing lines mounted on a frame, made it very difficult to establish a moiré setup in the lab. The moiré setup placed in IPRL used control parameters to record object curvatures. Using Takasaki’s equation [23], the height ($h_{n}$) between two curved patterns was calculated, which provided the nature of the surface curvatures. To allow exact photographic measurements of fringes on any object, a moiré contour graph was used. The problem of poor contrast occurred during test runs. It proved difficult to extract accurate curvatures of the object because of breaks in the curves.

## 5. Modified Moiré Fringe Topography Technique

**Figure 4:**
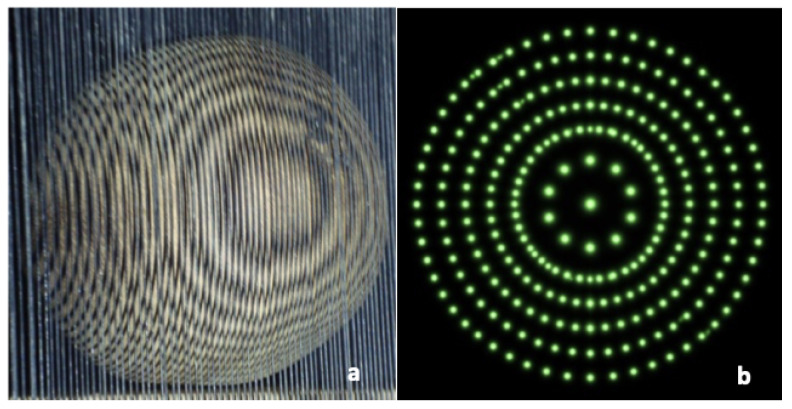
Results on the surface of a wooden sphere using (**a**) conventional and (**b**) modified moiré techniques.

In order to resolve this problem, the modified moiré technique is proposed which uses the circular dotted grid concept, as given in Figure 1e and Figure 2. It provides more accurate curvatures of the objects. Figure 4 shows the results of the conventional and modified moiré fringe topography results on the surface of a wooden sphere.

**Figure 5:**
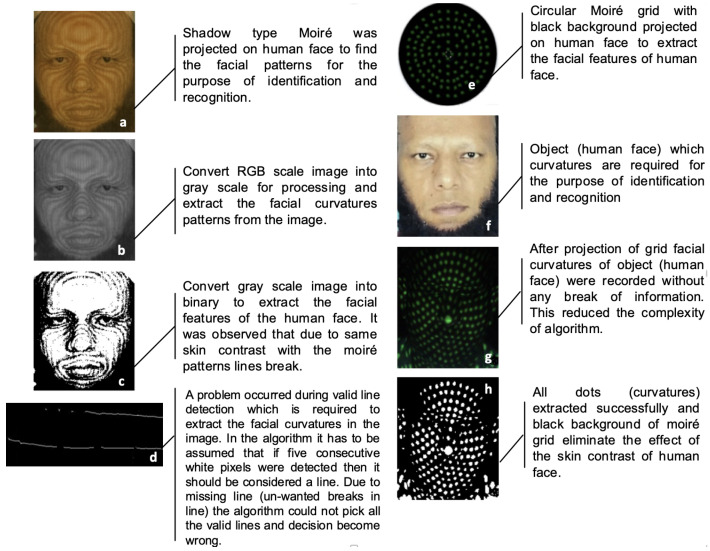
(**a**–**h**) Comparative results for conventional and dotted Moiré.

The proposed modification of using circular dotted-grid offers the following key advantages:

1. Improved Accuracy: Curvature estimation was more precise, as discrete circular dots provided clear reference points compared to continuous fringes that often merged or overlapped Figure 5c,d,g,h.
2. Enhanced Robustness: The use of circular dots minimized sensitivity to illumination variations, which are a common source of error in conventional moiré fringe topography Figure 5h.
3. Reduced Computational Complexity: Required computational complexity was lower due to the discrete point detection in the modified case, as compared with that required for the curve extraction and line tracing steps in the conventional case.

As explained through the results presented in the next section, the proposed circular dotted grid modification thus significantly improves the extraction of curvature information, enabling more accurate 3D surface representation of objects. This approach extends the applicability of moiré-based methods to complex biomedical and biometric analysis, where high precision is essential, and establishes the modified technique as a promising alternative for non-invasive human body surface analysis [5].

## 6. Experimental Results: Conventional and Modified Moiré Fringe Topography Technique

Experimental validation of both the conventional moiré fringe topography and the modified circular-dotted moiré technique was conducted at the IPRL. Three wooden objects with well-defined geometries such as a sphere, cylinder, and capsule were selected as test samples to provide measurable ground-truth curvature references.

### 6.1. Conventional Moiré Technique

In the case of conventional moiré fringe topography (wavy interference patterns [24]), curvature extraction was inconsistent due to fringe merging and poor contrast. As shown in Figure 4a, continuous fringes were often difficult to unwrap, resulting in errors in local phase order estimation.

### 6.2. Modified Circular-Dotted Moiré Technique

**Figure 6:**
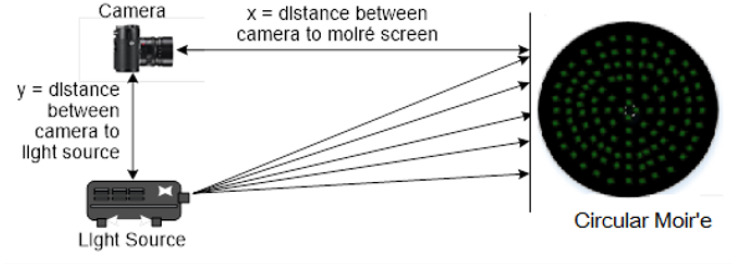
Experimental setup of modified projection system.

The proposed circular-dotted moiré approach overcame these limitations by projecting discrete circular markers instead of continuous fringes. This modification significantly enhanced contrast and robustness, as each dot served as a localized phase marker. The reconstructed surfaces from the dotted method were more consistent with ground-truth geometry and required less computational post-processing. The experimental setup of the modified projection system is shown in Figure 6.

#### 6.2.1. Quantitative Error Analysis

To evaluate accuracy, the reconstructed profiles of the sphere, cylinder, and capsule were compared against their physical dimensions. The Root Mean Square Error (RMSE) was computed as follows:

(9) $$RMSE=\sqrt{\frac{1}{N}\sum_{i=1}^{N}{\left(z_{i}^{measured}-z_{i}^{trust}\right)}^{2}}$$

where $z_{i}^{measured}$ is the reconstructed surface height, and $z_{i}^{t}rue$ is the ground-truth dimension at the same point.

Table 3 shows that the modified approach consistently reduced reconstruction error by 56–58% compared to the conventional method, in the case of inanimate objects, whereas for human faces, the reduction in error was 66.8%. This demonstrates that the circular-dotted moiré technique not only simplifies curvature detection but also significantly enhances quantitative accuracy. Extensive human-face testing is planned in the future.

Solved parameters: Table 4 presents the results of moiré fringe topography using conventional and modified techniques. The set parameters are given as follows:

(10) x=122cm,p=0.06cm,y=15cm

**Figure 7:**
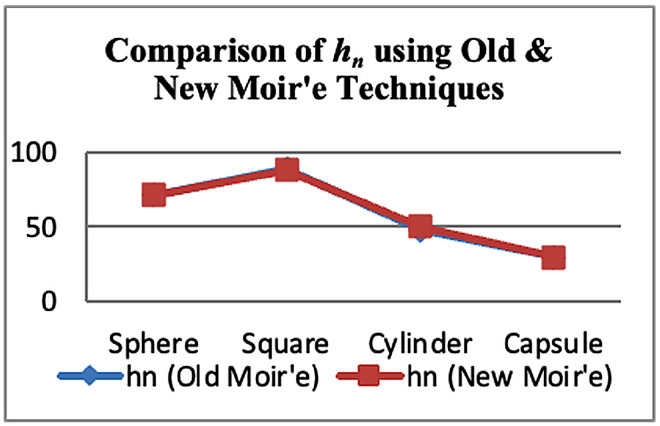
Comparison of solved parameters.

Figure 7 shows a comparison of solved parameters ($h_{n}$) of conventional and circular dotted moiré techniques. It is clear that circular dotted moiré techniques are consistent for surface screening.

#### 6.2.2. Generated Images

**Figure 8:**
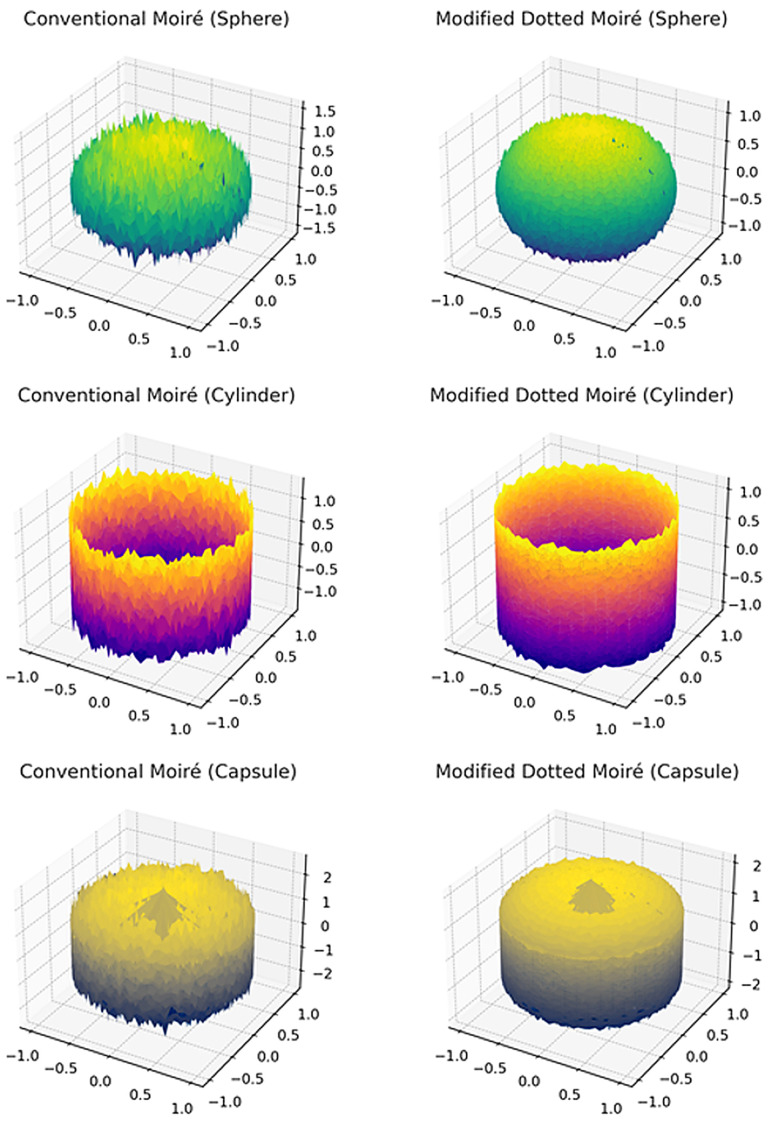
Creation of images using solved parameters for conventional and modified moiré techniques.

Figure 8 shows a graphical comparison of solved parameters ($h_{n}$) of conventional and circular dotted moiré techniques. Images were generated by the developed software using the solved parameters, such as $x=122\;\mathrm{cm},p=0.06\;\mathrm{cm},y=15\;\mathrm{cm},n_{h},n_{v}$ and $h_{n}$. It can be observed that images created using circular dotted moiré are consistent for surface screening and designing in 3D objects, and also more smooth compared with those generated using the conventional moiré technique.

#### 6.2.3. Accuracy, Precision and Specificity

The accuracy, precision and specificity of conventional line-based and dotted version of moiré fringe topography techniques for 30 samples (each of sphere, square, cylinder, capsule and human face) are summarized in Table 5. Each test run in IPRL is repeated three times to obtain better system accuracy and test repeatability.

It can be seen that the value of specificity has decreased. This reduced specificity does not undermine the reliability of the system for the surface diagnostics, for the following reasons:

1. *Screening-Oriented Application:* The proposed method is intended as a preliminary surface profiling or screening tool, where higher sensitivity and precision are prioritized to avoid missing true structural deviations. In such contexts, false positives are preferable to false negatives and can be resolved through secondary clinical evaluation.
2. *High Precision and Accuracy Preservation:* Despite the reduction in specificity, the method achieves significantly higher precision (96.70%) and accuracy (90.50%), indicating that detected positives are spatially consistent and geometrically accurate, which is critical for surface reconstruction reliability.
3. *False Positives Are Non-Critical and Controllable:* The false positives arise mainly from localized noise and texture-induced artifacts, not from systematic measurement errors. These can be effectively reduced through the following:
  – Adaptive thresholding;
  – Spatial filtering;
  – Morphological post-processing.

#### 6.2.4. Computational Complexity

Let us analyze and compare the computational complexity for each of the conventional and modified circular-dotted moiré techniques. The following assumptions are common to both techniques:

- Image resolution: N×M;
- Number of phase-shifted fringe images: *K* (typically 3–5);
- Phase unwrapping iterations: *I*;
- Number of detected dots in CDMT: *D*, where D.≪N×M

The algorithm steps for the conventional technique are as follows:

1. Project *K* phase-shifted fringe patterns;
2. Capture *K* images;
3. Image preprocessing (noise removal, normalization);
4. Pixel-wise phase calculation using phase-shifting equations;
5. Phase unwrapping (spatial/temporal);
6. Height/curvature reconstruction.

Here are the computational complexity values for each of these steps. Table 6 reports the complexity of the conventional technique, whereas Table 7 reports the corresponding complexity for the modified technique.

Thus, overall complexity for the conventional moiré technique is O(K.N.M+N.M.I)=O(N.M.(K+I)), where K≥3 and I≥1.

Its practical implications can be listed as follows:

- Global pixel-wise processing;
- High sensitivity to fringe breaks;
- Iterative phase unwrapping increases time;
- Computational load grows linearly with resolution and iterations.

Performing similar computational complexity analysis for the proposed modified technique, the algorithm steps are as follows:

1. Project single circular-dotted pattern;
2. Capture a single image;
3. Thresholding and dot segmentation;
4. Centroid detection for each dot;
5. Dot displacement analysis;
6. Surface reconstruction.

Thus, overall complexity for the modified circular-dotted moiré technique is O(N.M+D). Since D<<(N.M), the overall complexity becomes O(N.M).

Even when implemented without Fourier analysis, conventional moiré fringe topography relies on multi-frame phase-shifting and pixel-wise phase unwrapping, resulting in a computational complexity of O(N.M.(K+I)). In contrast, the modified circular-dotted moiré technique employs a single-shot dotted projection and centroid-based dot analysis, reducing the computational complexity to approximately O(N.M). This reduction explains the observed improvement in computational speed, robustness, and algorithmic simplicity of the proposed method.

#### 6.2.5. Specifications of the Experimental Setup

A Full-HD (1920 × 1080) DLP projector was used to project the green dotted pattern onto the object surface from a distance of approximately 1.2 m. Image acquisition was performed using a 5-megapixel CMOS camera positioned at an off-axis angle of 15°. This configuration ensured reliable centroid detection, minimal distortion, and accurate surface curvature reconstruction. The used dot sizes (digital size) in projector image were of radii of 3–7 pixels.

Here are the recommended experimental setup specifications for projector and camera, to avoid any calibration errors:

**Projector Specification:**

- Resolution: 1920 × 1080 (Full HD).
- Type: DLP or LED projector.
- Brightness: ≥2500 lumens.
- Pattern color: Green (λ≈520–540 nm).

*Justification:* Full HD resolution provides sufficient dot density for smooth curvature interpolation while avoiding aliasing. Green wavelength offers maximum camera sensitivity and contrast against human skin.

**Camera Specification:**

- Resolution: 1920 × 1080 (minimum) Preferred: 2592 × 1944 (5 MP).
- Sensor: CMOS.
- Frame rate: ≥30 fps.
- Lens: 25–35 mm (fixed focus recommended).

*Justification*: A minimum 1:1 or higher camera-to-projector resolution ensures accurate centroid localization and reduces quantization error in dot displacement measurement.

**Projection Distance:**

- Projector–Object distance: 1.0–1.5 m; Optimal: 1.2 m.
- Camera–Object distance: 1.2–1.8 m.
- Camera–Projector Angle: $10^{\circ }$–$20^{\circ }$ (off-axis).

*Justification:* This distance range balances the following:

- Dot size (∼1 mm on surface).
- Minimal perspective distortion.
- Comfortable subject positioning.
- Adequate depth sensitivity for facial curvature.

## 7. Conclusions and Discussion

I. Experimental trials at IPRL demonstrated that the circular-dotted moiré technique achieved reconstruction accuracy comparable to that of conventional moiré fringe topography across different wooden test objects, as summarized in Table 1. The proposed method consistently provided clearer curvature extraction, reduced post-processing complexity, and improved measurement robustness.

II. The primary advantage of the dotted moiré grid lies in its ability to transform continuous fringe patterns into discrete, high-contrast markers, thereby simplifying phase detection and reducing errors caused by fringe merging or illumination variations. This makes the technique not only easier to implement but also computationally more efficient, while maintaining quantitative accuracy.

III. Importantly, the proposed approach is not limited to wooden test objects only; results for human faces are shown in Table 3 and Table 4. Thus, this technique has strong potential for broader applications in fields where non-invasive, ionization-free surface screening is essential, such as biomedical diagnostics (e.g., scoliosis detection, facial morphology analysis), industrial metrology, and biometric recognition.

IV. Circular-dotted moiré-generated values are more consistent compared to line-based moiré values, as proven by the reduced values of coefficient-of-variation (Table 8) and circular, more smooth images (Figure 8).

V. Computational complexity analysis shows that the complexity of O(N.M.(K+I)) in the case of the conventional technique (where K+1≥4) is reduced to O(N.M) for the modified technique.

VI. Recommended experimental setup specifications for projector and camera have been suggested to avoid any calibration errors.

VII. At this initial stage of work, we have used the dark room for the experimentation with the modified technique. Performance under varying or adverse lighting conditions will be our future work.

VIII. In conclusion, the circular-dotted moiré technique represents a significant improvement over conventional fringe-based methods, offering a robust, accurate, and efficient framework for 3D surface reconstruction.

## Figures and Tables

**Table 1:** Comparison of conventional vs. modified raster-stereography.

Aspect	Conventional (Line-Based)	Modified (Dot-Based)
**Projection Pattern**	Continuous horizontal and vertical lines forming a grid	Dots at grid intersections
**Contrast Sensitivity**	High—lines break on textured/low-contrast surfaces	Low—dots remain visible even under poor contrast
**Feature Detection**	Requires curve tracing and line-following algorithm	Simplified centroid detection of dots
**Accuracy in Curvature**	Moderate—errors introduced by broken or overlapping lines	High—each dot provides an independent reference point
**Computational Complexity**	Higher due to curve extraction and line tracing steps	Lower due to discrete point detection

**Table 2:** Definition of Takasaki’s equation.

Parameters	Descriptions
**x**	Distance between camera and screen
**y**	Distance between light source and camera
**p**	Pitch of the moiré screen
**n**	Number of fringes

**Table 3:** Results of Root Mean Square Error (RMSE) values.

Object	Conventional Moiré RMSE (mm)	Modified Dotted Moiré RMSE (mm)	Improvement (%)
Sphere	2.15	0.92	57.2
Cylinder	1.84	0.76	58.7
Capsule	2.03	0.88	56.7
Human Face	2.95	0.98	66.8

**Table 4:** Result of Moiré Fringe Topography for both conventional (old) and modified (new) concepts.

S. No	Wooden Objects	Image of Objects	Objects (with Old Moiré Fringe)	Objects (with New Moiré Fringe)	No. of Patterns (Old Moiré)		Decision Parameter (Old Moiré)	No. of Patterns (New Moiré)		Decision Parameter (New Moiré)
					$n_{h}$	$n_{v}$	$h_{n}$ (cm) (×100)	$n_{h}$	$n_{v}$	$h_{n}$ (cm) (×100)
1	Sphere	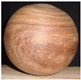	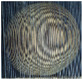	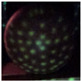	0.5040739	0.5035458	71.249642	0.5041427	0.5034216	71.2455731
2	Square	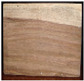	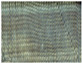	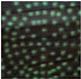	0.6224518	0.6341740	88.860728	0.6173421	0.6242615	87.7959959
3	Cylinder	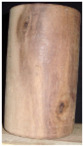	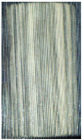	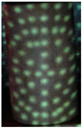	0.3312167	0.3452125	47.841004	0.3452389	0.3624790	50.0580586
4	Capsule	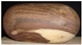	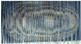	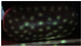	0.2120129	0.2103260	29.864108	0.2091368	0.2082881	29.5164588
5	Human Face	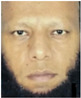	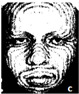	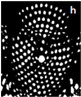	0.8122553	0.8211021	95.552102	0.8005412	0.8010246	94.857452
Total $h_{n}$ (cm)							333.36758			333.473538

**Table 5:** Accuracy, precision and persistence of moiré fringe topography techniques.

Parameters	New Moiré (Dotted-Based)	Conventional Moiré (Line-Based)
**Accuracy**	90.50	82.30
**Precision**	96.70	88.00
**Specificity**	35.30	52.00

**Table 6:** Computational complexity per step for the conventional technique.

Step	Complexity
Image preprocessing	O(K×N×M)
Phase calculation (arctangent per pixel) and labeling	O(N×M)
Phase unwrapping (iterative, neighborhood-based)	O(N×M×I)
Surface reconstruction	O(N×M)

**Table 7:** Computational complexity per step for the modified technique.

Step	Complexity
Image preprocessing	O(N×M)
Dot detection and labeling	O(N×M)
Centroid computation	O(D)
Displacement and geometry	O(D)
Surface reconstruction	O(D)

**Table 8:** Calculations of coefficient-of-variation values in conventional and dotted Moiré.

**1. Coefficient of variation in coordinate values of human faces with respect to x-deviations**
$$\mathrm{Coefficient}\mathrm{of}\mathrm{Variation}=\frac{\sigma }{\mu }\times 100$$ $$\sigma =\mathrm{Standard}\;\mathrm{Deviation}=\sqrt{\frac{\sum {\left(x_{i}-\mu \right)}^{2}}{N}}$$ $$\mu =\mathrm{Mean}=\frac{\sum x_{i}}{N}$$
After solving the parameters, we have
$$\mathrm{Coefficient}\mathrm{of}\mathrm{Variation}\left(\mathrm{Linebased}\mathrm{Moiré}\right)=\frac{\sigma }{\mu }\times 100=\frac{0.00621}{33.150}\times 100=0.018\%$$ $$\mathrm{Coefficient}\mathrm{of}\mathrm{Variation}\left(\mathrm{Dotted}\mathrm{Moiré}\right)=\frac{\sigma }{\mu }\times 100=\frac{0.0030}{35.250}\times 100=0.0085\%$$
**2. Coefficient of variation in coordinate values of a sphere with respect to x-deviations**
$$\mathrm{Coefficient}\mathrm{of}\mathrm{Variation}\left(\mathrm{Linebased}\mathrm{Moiré}\right)=\frac{\sigma }{\mu }\times 100=\frac{0.00050}{109.120}\times 100=0.00045\%$$ $$\mathrm{Coefficient}\mathrm{of}\mathrm{Variation}\left(\mathrm{Dotted}\mathrm{Moiré}\right)=\frac{\sigma }{\mu }\times 100=\frac{0.00013}{109.225}\times 100=0.00011\%$$

## Data Availability

The data presented in this study are available from the corresponding author upon reasonable request.
